# Links between electroconvulsive therapy responsive and cognitive impairment multimodal brain networks in late-life major depressive disorder

**DOI:** 10.1186/s12916-022-02678-6

**Published:** 2022-12-08

**Authors:** Shile Qi, Vince D. Calhoun, Daoqiang Zhang, Jeremy Miller, Zhi-De Deng, Katherine L. Narr, Yvette Sheline, Shawn M. McClintock, Rongtao Jiang, Xiao Yang, Joel Upston, Tom Jones, Jing Sui, Christopher C. Abbott

**Affiliations:** 1 College of Computer Science and Technology, Nanjing University of Aeronautics and Astronautics, Nanjing, China; 2 Tri-institutional Center for Translational Research in Neuroimaging and Data Science (TReNDS) Georgia State University, Georgia Institute of Technology, Emory University, Atlanta, GA USA; 3 Department of Psychiatry, University of New Mexico, Albuquerque, NM USA; 4 Noninvasive Neuromodulation Unit, Experimental Therapeutics & Pathophysiology Branch, National Institute of Mental Health, Bethesda, MD USA; 5 Departments of Neurology, Psychiatry and Biobehavioral Sciences, University of California, Los Angeles, CA USA; 6 Department of Psychiatry, University of Pennsylvania, Philadelphia, PA USA; 7 Division of Psychology, Department of Psychiatry, UT Southwestern Medical Center, Dallas, TX USA; 8 State Key Laboratory of Cognitive Neuroscience and Learning, Beijing Normal University, Beijing, China; 9 Huaxi Brain Research Center, West China Hospital of Sichuan University, Chengdu, China

**Keywords:** Electroconvulsive therapy, Antidepressant, Cognitive impairment, Electric field, Multimodal fusion

## Abstract

**Background:**

Although electroconvulsive therapy (ECT) is an effective treatment for depression, ECT cognitive impairment remains a major concern. The neurobiological underpinnings and mechanisms underlying ECT antidepressant and cognitive impairment effects remain unknown. This investigation aims to identify ECT antidepressant-response and cognitive-impairment multimodal brain networks and assesses whether they are associated with the ECT-induced electric field (E-field) with an optimal pulse amplitude estimation.

**Methods:**

A single site clinical trial focused on amplitude (600, 700, and 800 mA) included longitudinal multimodal imaging and clinical and cognitive assessments completed before and immediately after the ECT series (*n* = 54) for late-life depression. Another two independent validation cohorts (*n* = 84, *n* = 260) were included. Symptom and cognition were used as references to supervise fMRI and sMRI fusion to identify ECT antidepressant-response and cognitive-impairment multimodal brain networks. Correlations between ECT-induced E-field within these two networks and clinical and cognitive outcomes were calculated. An optimal pulse amplitude was estimated based on E-field within antidepressant-response and cognitive-impairment networks.

**Results:**

Decreased function in the superior orbitofrontal cortex and caudate accompanied with increased volume in medial temporal cortex showed covarying functional and structural alterations in both antidepressant-response and cognitive-impairment networks. Volume increases in the hippocampal complex and thalamus were antidepressant-response specific, and functional decreases in the amygdala and hippocampal complex were cognitive-impairment specific, which were validated in two independent datasets. The E-field within these two networks showed an inverse relationship with HDRS reduction and cognitive impairment. The optimal E-filed range as [92.7–113.9] V/m was estimated to maximize antidepressant outcomes without compromising cognitive safety.

**Conclusions:**

The large degree of overlap between antidepressant-response and cognitive-impairment networks challenges parameter development focused on precise E-field dosing with new electrode placements. The determination of the optimal individualized ECT amplitude within the antidepressant and cognitive networks may improve the treatment benefit–risk ratio.

**Trial registration:**

ClinicalTrials.gov Identifier: NCT02999269.

**Supplementary Information:**

The online version contains supplementary material available at 10.1186/s12916-022-02678-6.

## Background

Electroconvulsive therapy (ECT) is an effective treatment for major depressive disorder (MDD), especially in life-threatening and treatment-refractory conditions [1]. Although recognized for its unparalleled efficacy, ECT can also result in cognitive impairment, which prolongs recovery time, perpetuates the stigma associated with ECT, and deters patients and caregivers from this life-saving procedure [2]. While older age is associated with increased probability of response [3, 4], age is also associated with increased risk of ECT-mediated cognitive impairment [5]. The underlying neurobiological mechanisms of ECT antidepressant response and cognitive impairment actions remain elusive [6]. Prior research has suggested that a neuroplasticity restoration mechanism may contribute to the antidepressant action of ECT. A meta-analysis with 1728 adults with MDD and 7199 healthy adults has shown gray matter volume (GMV) reduction in the hippocampus, suggesting impaired neuroplasticity [7, 8], with another one showed disrupted functional brain topology [9]. Preclinical investigations have demonstrated increased cellular plasticity following electroconvulsive stimulations [10]. Older patients treated with right unilateral electrode placement have demonstrated lateralized medial temporal lobe neuroplasticity [11–13]. ECT-imaging meta-analyses demonstrated increased hippocampal volume after an ECT series that further substantiated neuroplasticity [14, 15]. ECT-imaging mega-analyses also showed increased hippocampal (*n* = 281) and whole-brain GMV (*n* = 328) [16, 17]. However, despite the larger sample sizes, these investigations found no associations between volumetric changes and antidepressant outcome.

Previous studies demonstrated that hippocampal-dependent cognitive functions, such as declarative memory, were most adversely affected by ECT [18]. In addition, working memory, verbal fluency, complex visual scanning, and cognitive flexibility can be adversely impacted following an acute ECT series [19]. Although cognitive impairment can be reduced by switching from bitemporal (BT) electrode placement with brief pulse width stimulation to right unilateral (RUL) electrode placement with ultrabrief pulse width [20], the latter still produces moderate to large (Cohen’s *d* = − 0.53 to − 0.83) adverse cognitive effects [21]. Recent studies have attempted to investigate cognitive correlates and brain volume changes, but these investigations were limited by small sample sizes (*n* < 25) and a focus only on the hippocampus [22, 23]. In this context, identifying the mechanisms and neuroanatomic locations of ECT-associated cognitive impairment is a critical step toward fostering research into ECT treatment parameters.

Historically, the electric stimulus has been viewed as the means to elicit seizure activity. However, specific ECT parameter settings (e.g., RUL at seizure threshold, BT electrode placement with ultrabrief pulse width, low amplitude ECT) generate seizure activity but have limited antidepressant benefit. While a seizure is generally considered necessary for ECT-induced antidepressant effects, electrical stimulation also plays a critical role in antidepressant and cognitive outcomes. With respect to ECT parameters, the electrode geometry and placement determines the spatial distribution of the induced electric field (E-field), and the current pulse amplitude determines the E-field magnitude of this spatial distribution [24]. Spherical head models have demonstrated that the E-field is influenced by individual anatomic variability (skull thickness, head diameter, and brain volume) [24, 25]. Anatomical-realistic E-field models are based on a patient’s structural MRI (sMRI), accounting for individual variation in head and brain anatomy and their influence on the induced current flow [26]. Using patient-specific models, the ECT-induced regional E-field strength has been shown to be associated with structural changes and antidepressant outcomes [27–29]. Computer modeling has also demonstrated the importance of age-related brain changes in relation to electric field strength and pulse amplitude. Specifically, pulse amplitude must increase to maintain the same volume of stimulated neuronal volume due to brain atrophy [25, 30].

Recent investigations have applied data-driven, whole brain analysis methods to identify ECT antidepressant-response networks. A linear classifier (linear discriminant analysis) demonstrated that antidepressant-responsive structural changes extend beyond the hippocampus and are widely distributed in cortical and subcortical regions [31]. A multimodal fusion analysis showed that ECT response is associated with reduced fractional amplitude of low frequency fluctuations (fALFF, measures the local spontaneous neuronal activity that improves the sensitivity and specificity of detecting regional functional brain activities) in the prefrontal cortex and hippocampus accompanied with increased GMV in medial temporal cortex (MTC), thalamus, and hippocampus, which were unassociated with ECT-associated cognitive impairment [32]. These results suggest that ECT treatment responsive and cognitive impairment networks are separate.

The present study used supervised fusion with structural and functional imaging to identify ECT antidepressant-response and ECT cognitive-impairment networks in a sample of older ECT patients. For this investigation, we focused on the following aims: (1) identify ECT antidepressant-response multimodal brain network, (2) identify ECT cognitive-impairment network, (3) compare the ECT antidepressant-response and cognitive-impairment networks, (4) validate the identified antidepressant-response and cognitive-impairment networks in two independent data sets, (5) evaluate the relationship between ECT E-field within these two networks with clinical and cognitive outcomes, and (6) determine the optimal pulse amplitude estimation based on antidepressant and cognitive networks.

## Methods

### Participants

Older adult patients with MDD (*n* = 54) investigated in this study were recruited from the University of New Mexico (UNM) from December 2016 to September 2019. Two independent psychiatric evaluations were performed to confirm diagnosis of MDD (non-psychotic or psychotic episodes, single episode or recurrent) and the clinical indication for ECT. Additional inclusion criteria included right-handedness and age (50–80, which is associated with ECT-induced cognitive impairment [5, 33] and an increased probability of antidepressant response [4]). Exclusion criteria included neurological or neurodegenerative disorder (e.g., head injury, epilepsy, or Alzheimer’s disease), other psychiatric disorders (e.g., bipolar disorder, schizoaffective disorder, and schizophrenia), substance (except nicotine) or alcohol use disorder, and contraindications to MRI. To reduce medication confounds, all subjects tapered and discontinued their scheduled antidepressant medications prior to the baseline assessment, but as-needed medications were permissible for anxiety and insomnia including trazodone (maximal cumulative dose per day: 200 mg), lorazepam (3 mg), and quetiapine (200 mg). Patients provided written informed consent after receiving a complete description of the study, which was approved by UNM Human Research Protections Office.

All patients completed the cognitive, clinical, and imaging assessments on the day before and within 1 week of finishing the acute ECT series. Multimodal fMRI and sMRI data were collected and preprocessed to fALFF and GMV as fusion input. Details on imaging parameters and preprocessing steps can be found in Additional file 1: “Imaging parameters and preprocessing” and “Head Motion” sections. Note that global signal regression was applied in the preprocessing of fMRI in order to remove head motion, cardiac, and respiratory signals known to correlate with the global signal [34, 35]. Processing speed, verbal fluency, inhibition, and cognitive flexibility were measured by the Delis Kaplan Executive Function System (DKEFS), specifically the Verbal Fluency Test [36]. Among all the cognitive measures, the DKEFS Verbal Fluency Letter Fluency Total Correct Scaled Score (DKVFLFSS) had the most significant longitudinal difference between pre- and post-ECT, i.e., the highest cognitive decline after the ECT series [37]. Premorbid function was measured with the Test of Premorbid Function [38]. The Hamilton Depression Rating Scale (HDRS) 24-item was used as the primary depression rating scale. The demographic and clinical information are summarized in Table 1. The longitudinal differences between pre- and post-ECT reflected antidepressant response (*p* = 1.2e−15 for HDRS) and cognitive impairment (*p* = 6.8e−06 for DKVFLFSS).

**Table 1:** Demographic and clinical information of discovery ECT1 cohort

	MDD	*p*1^#^	*p*2^##^
**Demographic characteristics**			
Sample size (*n*)	*n* = 54		
Age (years)	65.4 ± 8.8	0.20	0.20
Sex (M/F)	16/38	0.57	0.20
Ethnicity (Non-Hispanic/Hispanic)	46/8	0.32	0.97
Race (Caucasian/African American/Hispanic/Asian)	46/1/6/1	0.10	0.21
Education degree**	5.3 ± 1.8	0.82	0.19
Handiness (R/L)	54/0	n/a	n/a
Height	1.7 ± 0.1	0.19	0.19
Weight	72.3 ± 20.1	0.12	0.13
BMI	25.9 ± 5.9	0.25	0.27
IQ	109.8 ± 1	0.58	0.29
Mean FD (PRE)	0.25 ± 0.12	0.82	0.96
Mean FD (POST)	0.27 ± 0.14	0.55	0.90
**Clinical characteristics**			
Age onset	36 ± 19.9	0.26	0.11
Age treated	40.4 ± 17.5	0.99	0.19
Single episode/recurrent	6/48	0.56	0.62
Number of major depressive episodes	4.1 ± 4.1	0.49	0.04
Duration of current depressive episode (months)	17.5 ± 22.1	0.86	0.21
Lifetime duration years (years)	7.5 ± 10.4	0.59	0.47
Total number of ECT treatments	10.8 ± 3.3	0.42	0.14
Maudsley scale for treatment resistance	8.9 ± 2.0	0.41	0.90
Last treatment: RUL/BT*	33/21	0.76	0.11
ΔHDRS	19.9 ± 13.0	0.76	0
ΔDKVFLFSS	5.4 ± 9.6	0	0.76

*RUL, right unilateral; *BT* bitemporal

**“Education degree” details are presented in Additional file 1: “Education degree” section

^#^“*p*1” denotes the *p* values for the correlation between ΔHDRS

^##^“*p*2” denotes the *p* values for the correlation between ΔDKVFLFSS

A ± B represents mean ± standard deviation

Δ means PRE-POST

*FD* frame-wise displacement

All patients started with RUL electrode placement and were randomized and blinded to 600, 700, and 800 mA prior to the first ECT treatment with either 0.3 or 1.0 millisecond (ms) pulse width (brief pulse width adopted towards latter part of the study with an attempt improve antidepressant response rates in the low amplitude arm). Individualized seizure thresholds with subsequent treatments at six times seizure threshold determined other parameters (pulse train duration and frequency). If patients were non-responsive to the assigned amplitude (< 25% reduction in HDRS at mid-ECT evaluation), BT electrode placement with 800 mA and 1.0 ms pulse width was used for the remainder of the ECT series. Patients received general anesthesia with methohexital and succinylcholine. ECT clinicians determined the ECT treatment number based on clinical judgment. Clinical outcomes and details of the ECT administration have been previously reported (ClinicalTrials.gov Identifier: NCT02999269 [39]).

The ECT-induced E-field was computed with the Simulation of Non-Invasive Brain Stimulation (SimNIBS) software [40]. Electrodes were added to the head mesh in either RUL or BT orientation. SimNIBS then calculated the E-field throughout the head mesh and resampled E-field distribution map to voxel resolution. The initial E-field map was created with 1 mA current with either a RUL or BT orientation. The input current was used as a multiplier to determine the final E-field strength (600, 700, or 800 mA, details can be found in Additional file 1: “Electric field” section). The demographic and clinical information comparison among 600, 700, and 800 mA groups are summarized in Additional file 1: Table S1. The number of depressive episodes was correlated with ΔDKVFLFSS (Table 1) and was regressed out from the whole brain fALFF and GMV feature matrices (dimension = patient number multiplied by 53*63*46) prior to the fusion with reference analysis.

### Validation datasets

A second dataset included MDDs receiving ECT with pre-/post-ECT multimodal imaging (*n* = 84, Additional file 1: Validation1, independent ECT dataset [32]) was used to validate the treatment responsiveness of the identified ECT antidepressant-response and cognitive-impairment networks [41]. MDDs were recruited from the University of California Los Angeles (UCLA) and UNM after meeting the clinical indication for ECT. Patients completed the cognitive testing on the same date as MRI scans. Pre-ECT scans were completed within 2 days of ECT start and post-ECT assessment completed within 7 days of finishing ECT series. Linear projection of the identified antidepressant/cognitive-impairment networks (the discovery dataset) to an independent ECT dataset (Validation1 dataset) was performed to test whether the ECT responsiveness of these networks can be replicated.

A third dataset included MDDs with no ECT treatment intervention (*n* = 260, Additional file 1: Validation2, independent baseline MDD dataset [42]) was used to further verify the relationships between shared regions of antidepressant-response and cognitive-impairment networks with depression severity/cognition cross-sectionally. Specifically, the common brain regions in ECT-responsive and cognitive networks (the discovery dataset) were used as ROIs to extract the brain features from the baseline MDDs (Validation2 dataset). Correlation analysis was performed between the common ROIs with clinical scores within validation2 MDDs to show whether ECT-responsive and cognitive-impairment networks are really associated with symptom and cognition in baseline MDDs.

### Analysis pipeline

**Fig. 1:**
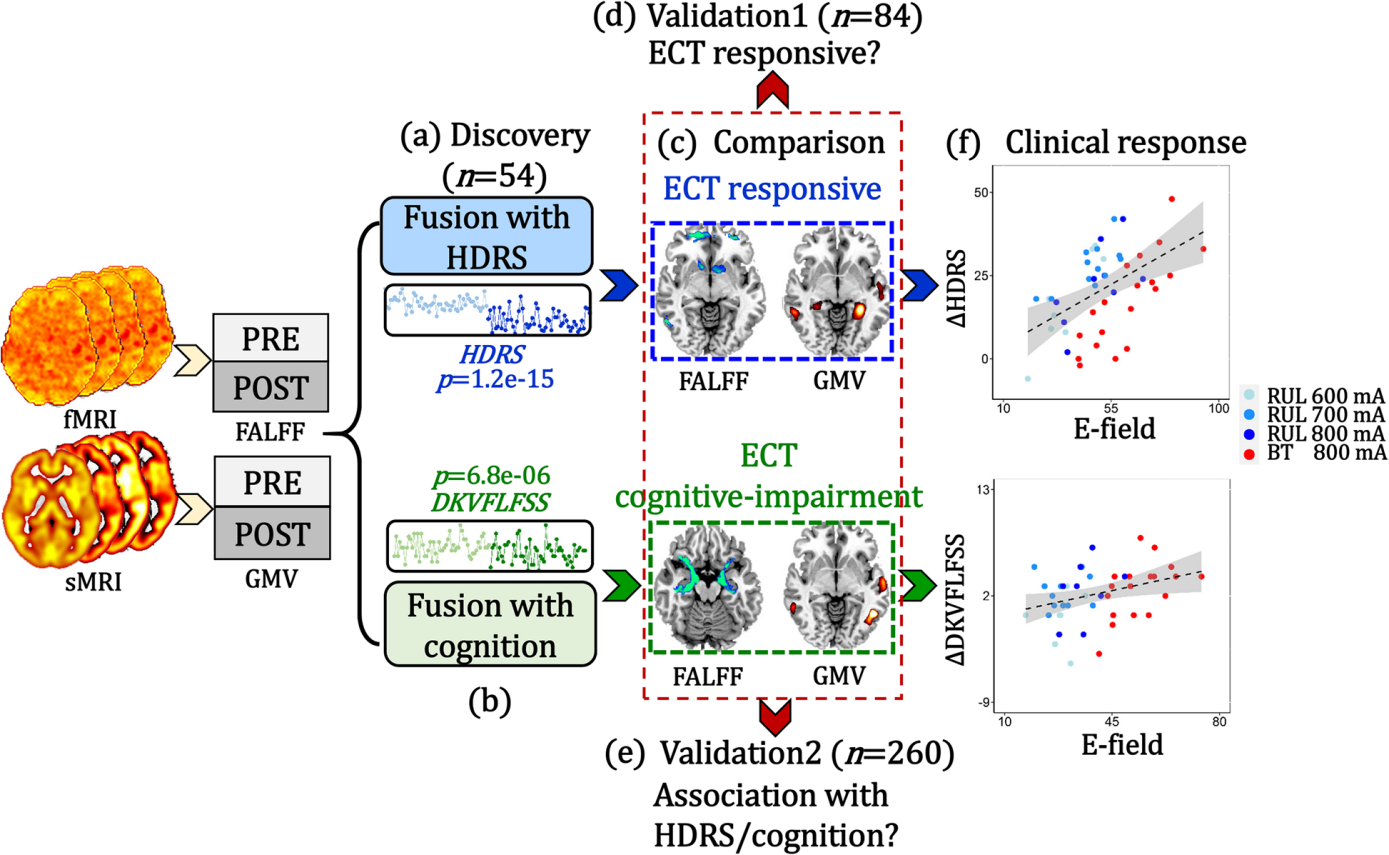
Flow diagram of the study design. **a** HDRS-guided fusion was performed on fALFF+GMV to identify ECT antidepressant-response multimodal brain network. **b** DKVFLFSS-guided fusion was performed on fALFF+GMV to identify ECT cognitive-impairment multimodal brain network. The *p*-values in **a**–**b** represent the longitudinal group difference of HDRS and DKVFLFSS. **c** Comparisons between antidepressant-response and cognitive-impairment networks identified common and specific regions between these two networks. **d** Linear projection projected the identified antidepressant-response and cognitive-impairment components onto an independent second ECT dataset for validation (*n* = 84). **e** Averaged fALFF/GMV was calculated within the common areas between antidepressant-response and cognitive-impairment networks to test whether the common regions are associated with depression severity/cognition with a third independent dataset (*n* = 260). **f** The associations between the ECT-induced E-field within these two networks and clinical responses were assessed. **g** Optimal pulse amplitude was estimated based on receiver operating characteristic curve analysis for both antidepressant and cognitive impairment networks

**Fig. 2:**
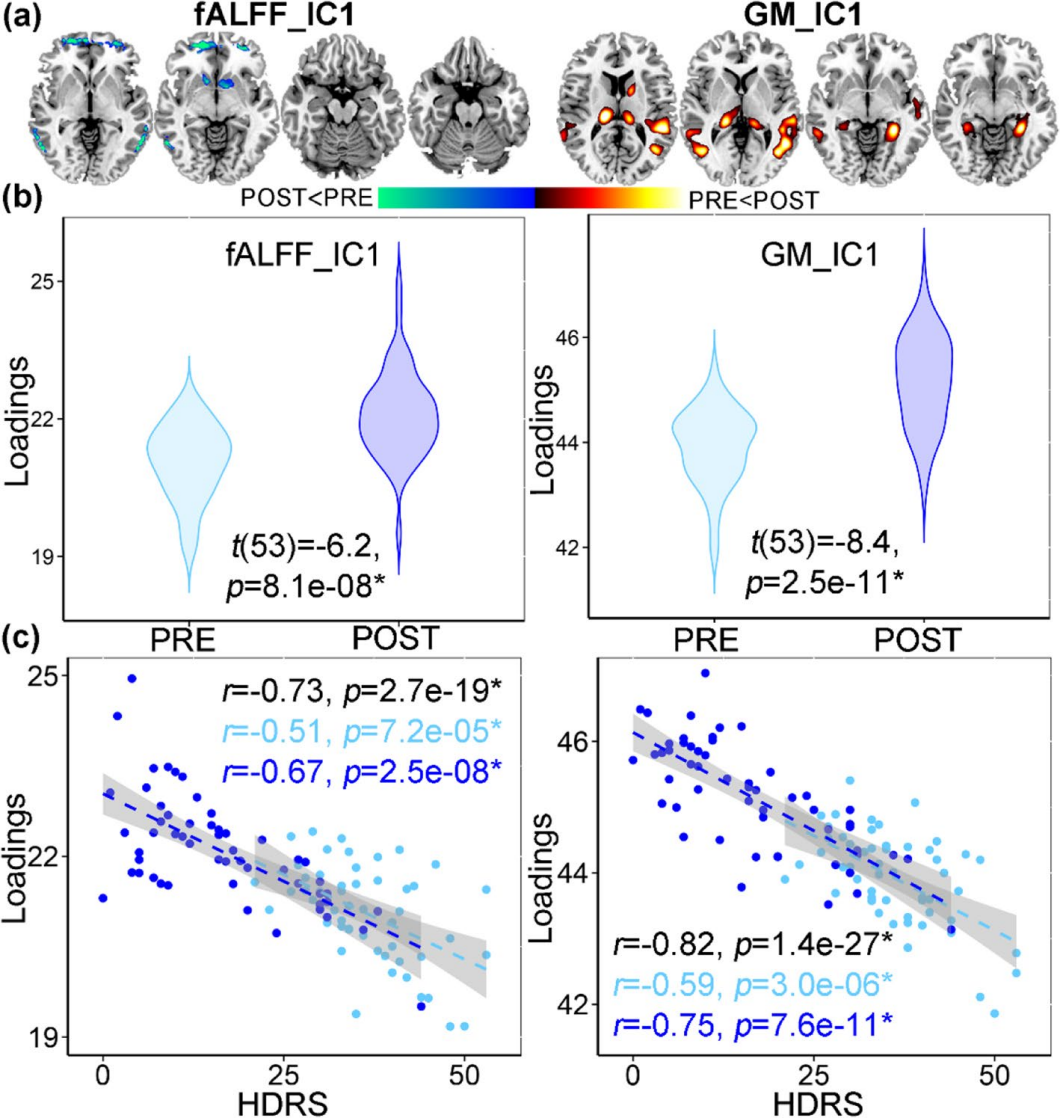
ECT antidepressant-response fALFF+GMV multimodal joint components. **a** The spatial brain networks visualized at |Z|>2.5. **b** Longitudinal PRE and POST ECT difference of the loadings (contribution weight of the extracted network across patients). **c** Correlation between components’ loadings and HDRS. The cyan and blue lines represent correlation within PRE and POST groups, respectively. The brain areas (Fig. 2a) in ECT responsive network were summarized in Additional file 1: Table S2 for fALFF and GM (Talairach labels), respectively. Loadings represent the patient-wise contribution weights of the corresponding component

**Fig. 3:**
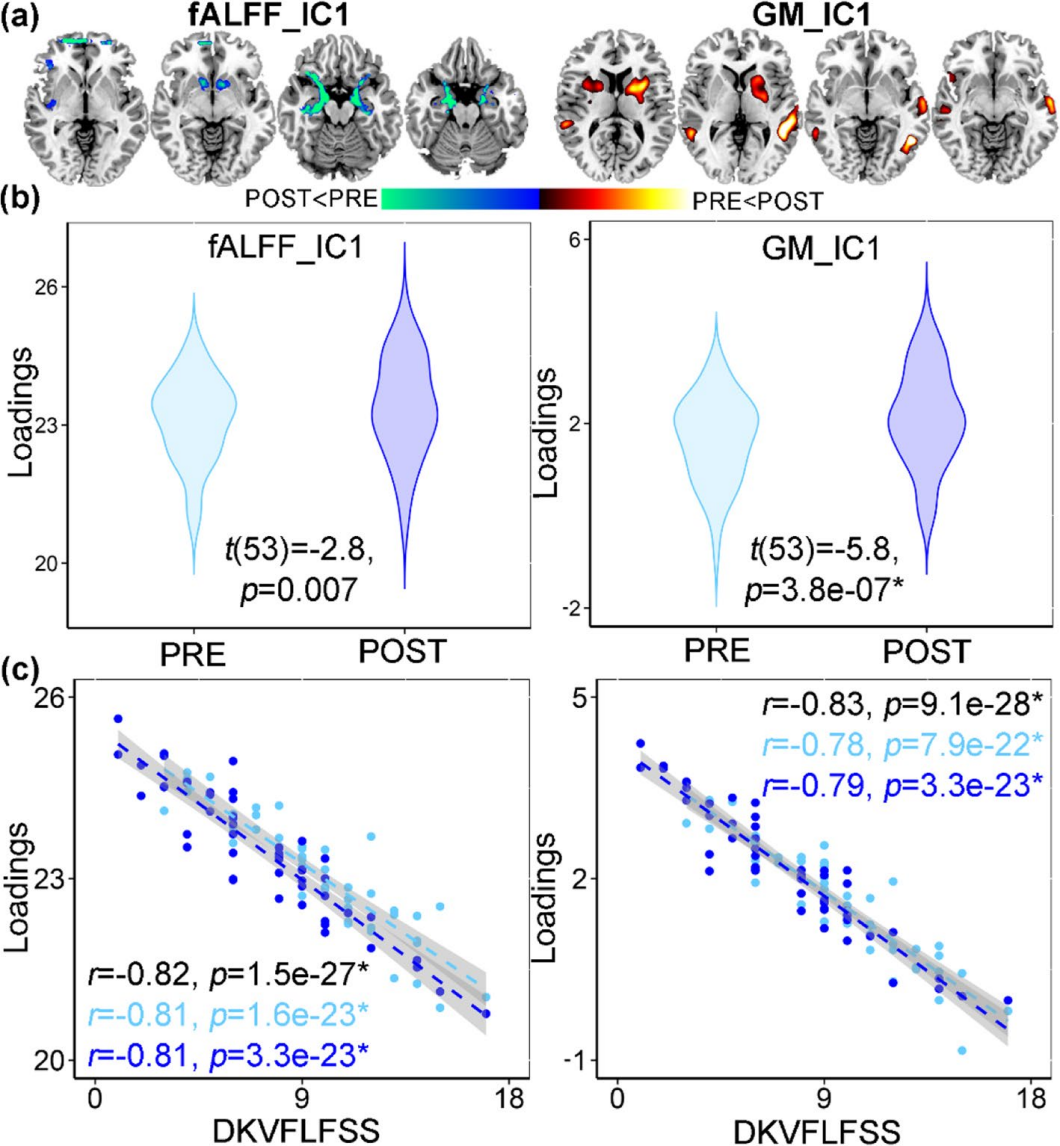
ECT cognitive-impairment fALFF+GMV multimodal joint components. **a** The spatial brain networks visualized at |Z|>2.5. **b** Longitudinal PRE and POST ECT difference of the loadings. **c** Correlation between components’ loadings and Delis Keplin Executive Functioning Letter Fluency Scaled Score (DKVFLFSS). The cyan and blue lines represent correlation within PRE and POST ECT, respectively. The brain areas (**a**) in the ECT-associated cognitive impairment network were summarized in Additional file 1: Table S3

**Fig. 4:**
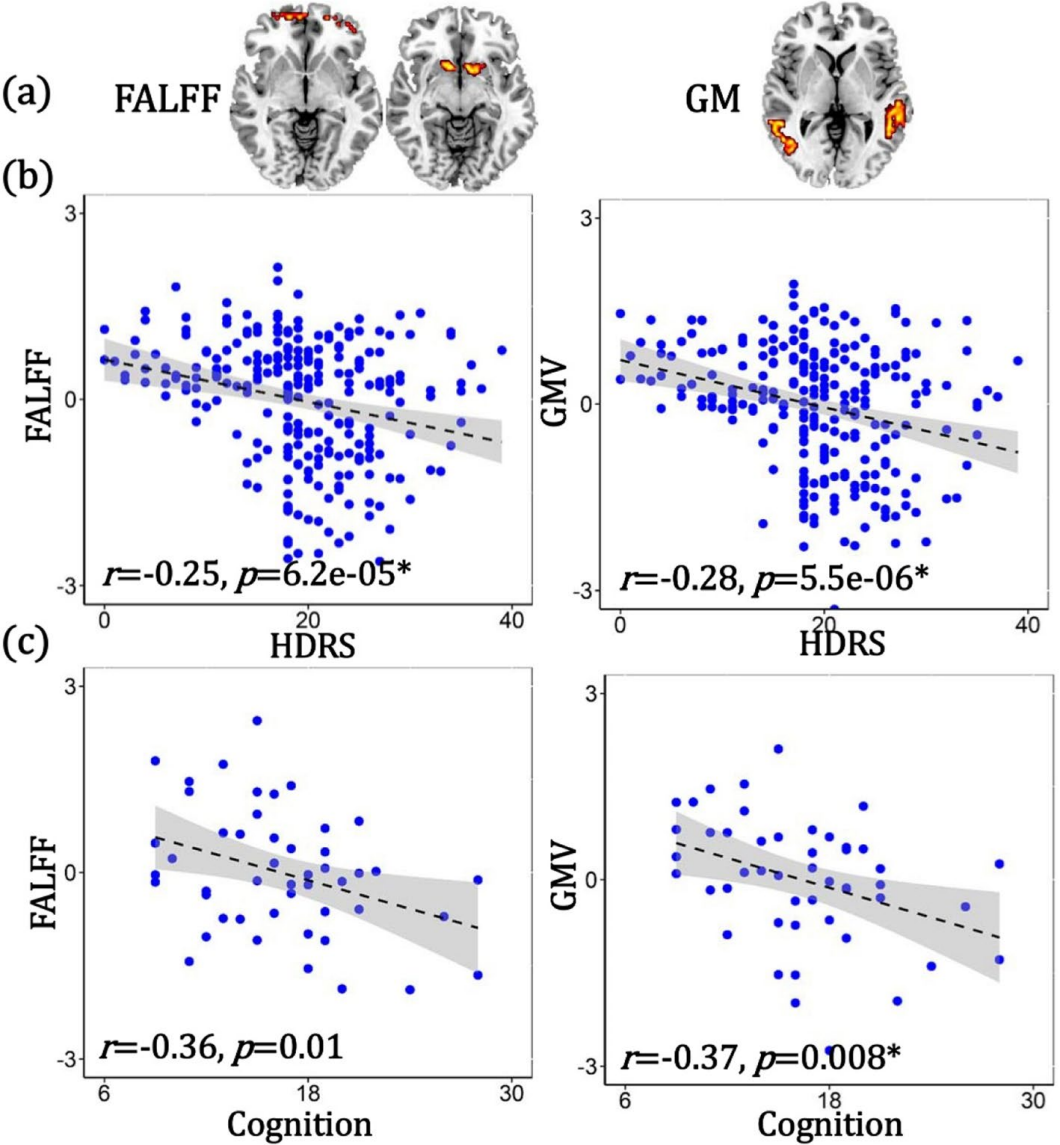
The identified common areas (**a**, overlapping between ECT antidepressant and cognitive-impairment networks) were correlated with both HDRS **b** and cognition **c** in independent baseline MDD dataset

**Fig. 5:**
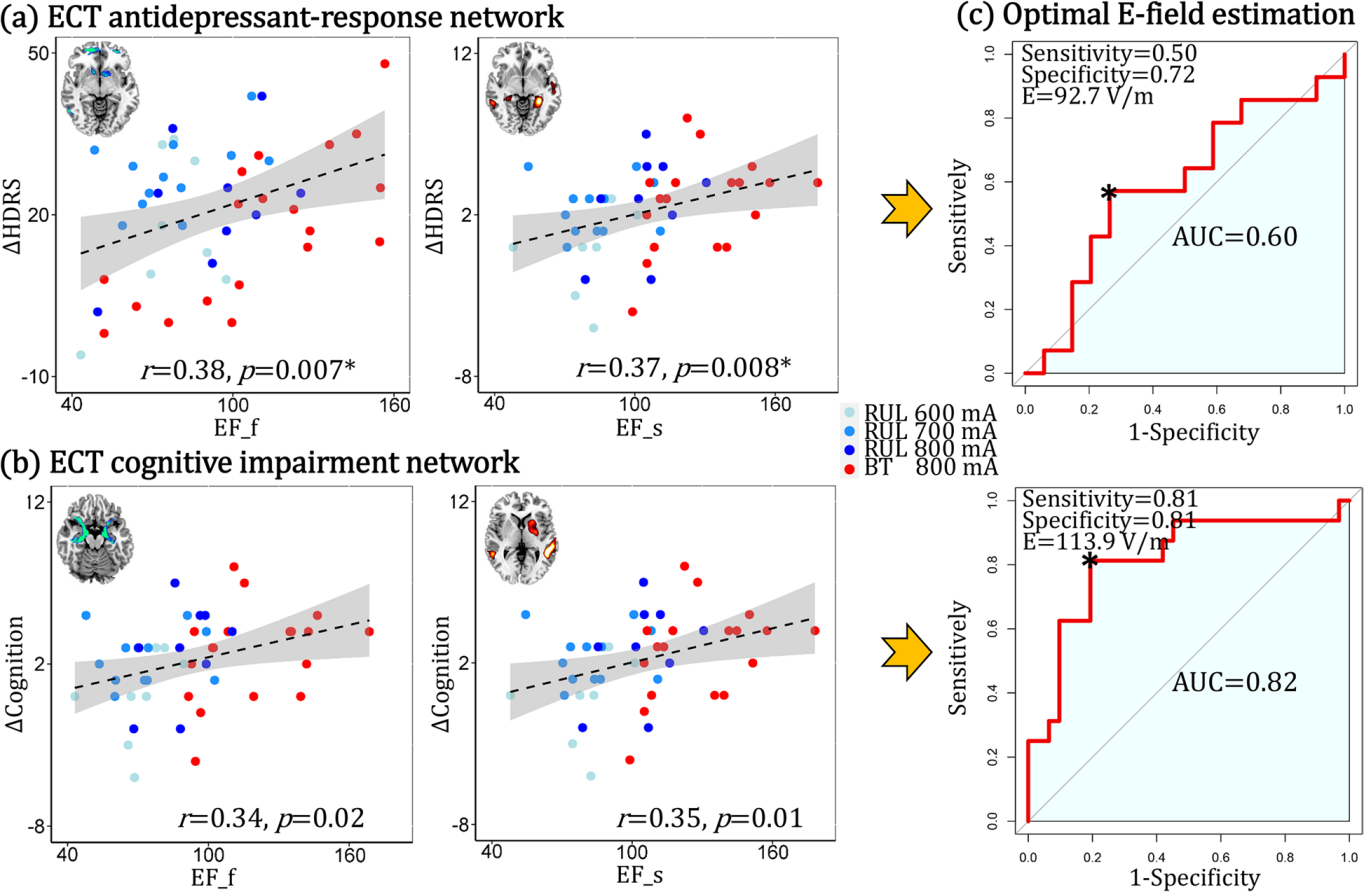
**a** Correlation between ∆HDRS and the mean E-field within ECT antidepressant-response network. **b** Correlation between ∆DKVFLFSS and the mean E-field within ECT cognitive-impairment network. EF_f and EF_s represent E-field within functional and structural brain networks. **c** Optimal pulse amplitude estimation from both antidepressant-response and cognitive impairment networks

According to the goals stated in the introduction, we performed the following analyses: (1) HDRS-guided fusion was used to identify ECT antidepressant-response multimodal brain network (Fig. 1a); (2) DKVFLFSS-guided fusion was used to identify ECT cognitive-impairment multimodal brain network (Fig. 1b); (3) antidepressant-response and cognitive-impairment networks were compared to identify common and specific brain regions (Fig. 1c); (4) antidepressant-response and cognitive-impairment networks were validated within independent datasets (Fig. 1 d–e); (5) the relationship between ECT-induced E-field and clinical responses were compared with correlational analyses (Fig. 1 f); and (6) optimal pulse amplitudes were estimated based on receiver operating characteristic (ROC) curve analysis (Fig. 1 g).

Specifically, patient-wise HDRS/DKVFLFSS scores were used as a reference to jointly decompose the preprocessed fALFF and GMV by “MCCAR+jICA” [43–46] (multi-site canonical correlation analysis with reference + joint independent component analysis) to investigate the antidepressant-response and cognitive-impairment fALFF-GMV covarying multimodal brain networks (Fig. 1 a, b). This fusion with reference model can maximize the correlations of imaging components with clinical measures of interest (i.e., HDRS/DKVFLFSS), as in Eq. (1).

1 $$\max \sum\nolimits_{\textrm{k},\textrm{j}=1}^2\left\{{\left\Vert \textrm{corr}\left({\textrm{A}}_{\textrm{k}},{\textrm{A}}_{\textrm{j}}\right)\right\Vert}_2^2+2\uplambda \bullet {\left\Vert \textrm{corr}\left({\textrm{A}}_{\textrm{k}},\textrm{ref}\right)\right\Vert}_2^2\right\}$$

where A_k_ is the mixing matrix for each modality, corr(A_k_, A_j_) is the column-wise correlation between A_k_ and A_j_, and corr(A_k_, ref) is the column-wise correlation between A_k_ and the reference signal (HDRS/DKVFLFSS). This supervised fusion method can extract a joint multimodal component(s) that correlate with HDRS/DKVFLFSS.

For validation, linear projection projected the spatial maps of discovery ECT onto an independent ECT-imaging data set. An estimation of mixing matrix for each modality was obtained that can be used to verify whether these two networks were associated with antidepressant and cognitive outcomes (Fig. 1d, Additional file 1: “Linear projection”). Comparing the ECT antidepressant-response (HDRS associated) and cognitive-impairment (DKVFLFSS associated) networks identified common and unique patterns associated within each network (Fig. 1c). These common areas were used as ROIs to calculate the average fALFF/GMV over all the patients in another independent data set to assess the relationship between shared regions of the antidepressant-response and cognitive-impairment networks and depression severity/cognition (Fig. 1e).

The 90th percentile of E-field magnitude from all voxels was calculated for each patient within these networks (ROIs), serving as an estimate of the peak induced field strength while avoiding the influence of tissue boundary effects that could bias the absolute maximum E-field values. Correlation analysis was performed to evaluate the relationship between ECT-induced E-field within antidepressant-response and cognitive-impairment networks and clinical responses (including ∆HDRS and ∆DKVFLFSS, Fig. 1f). Finally, an optimal pulse amplitude was estimated from the ROC curve for the binary classification (Fig. 1g) based on E-field within antidepressant and cognitive-impairment networks, in which the lower bound of test-retest reliability of DKVFLFSS was -3 (class one: DKVFLFSS>-3; class two: DKVFLFSS<=-3) and ECT response was defined as >50% improvement from baseline in HDRS.

## Results

### ECT antidepressant-response multimodal brain network

HDRS-associated multimodal component was identified (the first independent component, IC1, Fig. 2a) that longitudinally discriminated (paired *t*-tests) between PRE- and POST-ECT (Fig. 2b, for fALFF, *t*(53) = − 6.2, *p* = 8.1e−08*, power = 0.99; for GMV, *t*(53) = − 8.4, *p* = 2.5e−11*, power = 1, Additional file 1: “Power analysis”). The (*) signifies statistical significance with a false discovery rate (FDR) correction for multiple comparisons. The loadings (contribution of weight of the extracted network across patients) of ECT responsive components were negatively correlated with HDRS scores (for fALFF, *r* = − 0.73, *p* = 2.7e−19*; for GMV, *r* = − 0.82, *p* = 9.1e−27*, Fig. 2c). This negative association was observed when looking separately at each time point (PRE and POST) as well as both time points together and remained significant even after regressing out the number of ECT sessions (for fALFF, *r* = − 0.63, *p* = 3.2e−15*; for GMV, *r* = − 0.76, *p* = 8.1e−20*). After the ECT series, decreased fALFF in superior orbitofrontal cortex (SOFC) and caudate were accompanied with increased GMV in MTC, thalamus, parahippocampus. and hippocampus.

### ECT cognitive-impairment network

A cognition-associated multimodal component (Fig. 3a) was identified that longitudinally discriminated between PRE- and POST-ECT (Fig. 3b, for fALFF, *t*(53) =-2.8, *p* = 0.007, power = 0.6; for GMV, *t*(53) = − 5.8, *p* = 3.8e−07*, power = 0.7). The loadings of the cognitive-impairment network were negatively correlated with the DKVFLFSS scores (for fALFF, *r* = − 0.82, *p* = 1.5e−27*; for GMV, *r* = − 0.83, *p* = 9.1e−28*, Fig. 3c). This negative association was observed when looking separately at each time point (PRE and POST) as well as both time points together and remained significant after regressing out the number of ECT sessions and premorbid IQ (for fALFF, *r* = − 0.71, *p* = 1.4e−22*; for GMV, *r* = − 0.73, *p* = 8.1e−23*). After the acute ECT series, decreased fALFF in the SOFC, caudate, hippocampus, parahippocampus, and amygdala were accompanied with increased GMV in the MTC and putamen. The identified antidepressant-response and cognitive-impairment networks were unrelated to the number of ECT sessions (Additional file 1: Table S4). The identified ECT-antidepressant and cognitive-impairment networks in the discovery cohort remained ECT responsive and were correlated with HDRS in independent ECT dataset (Additional file 1: Fig. S1-2), demonstrating the replicability of ECT-responsiveness.

### Comparison between antidepressant-response and cognitive-impairment networks

When comparing the ECT antidepressant-response and cognitive-impairment networks (Additional file 1: Fig. S3a-b), we found that decreased fALFF in the SOFC and caudate accompanied with increased GMV in MTC were the common covarying functional and structural alterations after ECT treatment. Furthermore, GMV increase in the hippocampus, parahippocampus, and thalamus was unique in the ECT antidepressant-response network, and fALFF decrease in the amygdala, hippocampus, and parahippocampus was unique in the ECT cognitive-impairment network. All the above identified similarities and differences between antidepressant-response and cognitive-impairment networks were replicated on the subset of those patients treated with only RUL electrode placement (*n* = 33, Additional file 1: Fig. S3c-d). The identified common areas between antidepressant and cognitive-impairment networks were found to be correlated with both depression severity and cognition in independent cross-sectional MDD dataset (Fig. 4), demonstrating the associations between the common regions with symptom and cognition in baseline MDDs.

### Relationship between ECT-induced E-field and clinical responses and optimal pulse amplitude estimation

The E-field within the ECT antidepressant-response network was positively correlated with ∆HDRS (PRE-POST), i.e., the higher E-field the lower the POST HDRS (Fig. 5a). The E-field within the cognitive-impairment network was positively correlated with the ∆DKVFLFSS (cognitive decline), i.e., the higher E-field the lower POST cognition (Fig. 5b). More importantly, ROC for E-field within antidepressant/cognitive networks revealed an area under the curve (AUC) of 0.60/0.82 and maximal sensitivity and specificity of 92.7/113.9 Volts/meter (V/m) (Fig. 5c). So, the optimal E-filed range as [92.7–113.9] V/m was estimated to maximize antidepressant outcomes without compromising cognitive safety.

## Discussion

In this data-driven, longitudinal investigation, we identified ECT antidepressant-response and ECT cognitive-impairment multimodal networks in late-life MDDs. The strengths of this study included the data driven, multi-modal fusion to investigate and compare antidepressant and cognitive impairment networks, which were validated in independent datasets. This study demonstrated the following results. First, the ECT antidepressant-response network was associated with decreased fALFF in SOFC and caudate accompanied with increased GMV in hippocampal complex, thalamus, and MTC. Second, the ECT cognitive-impairment network was associated with decreased fALFF in SOFC, caudate, hippocampal complex, and amygdala covaried with increased GMV in putamen and MTC. Third, comparing antidepressant-response and cognitive-impairment networks revealed network overlapping (decreased fALFF in SOFC and caudate accompanied with increased GMV in MTC) and specific changes (increased GMV in hippocampal complex and thalamus is unique to the antidepressant network; decreased fALFF in hippocampal complex and amygdala is specific to the cognitive impairment network). Fourth, increased E-field strength within these two networks have opposite effects with improved antidepressant outcomes and compromised cognitive safety. Fifth, the optimal pulse amplitude to improve both antidepressant response and cognitive safety in ECT was estimated as [92.7–113.9] V/m. In the following discussion section, we contextualize these findings in the context of ECT parameter development with the identification of targets (ECT antidepressant-response biomarkers) and anti-targets (ECT cognitive-impairment biomarkers).

ECT antidepressant-response biomarkers are neuroanatomic targets for stimulation. A major finding was the identification of ECT antidepressant-response multimodal brain networks. Our previous investigation with an independent multi-site dataset and the same data-driven multi-fusion methodology demonstrated remarkable similarity with reduced fALFF in the SOFC and increased GMV in the hippocampal complex, thalamus, and MTC [32]. In contrast, Global ECT MRI Collaboration (GEMRIC) study identified sMRI antidepressant-responsive regions with modest overlap in the MTC and parahippocampus with notable absence of hippocampal volumetric changes [31]. Mechanistically, these studies converge in demonstrating that ECT antidepressant mechanisms are related to neuroplasticity in specific anatomic regions. Key differences between these studies include the use of multi-modal fusion (sMRI and rs-fMRI) in the present investigation to identify treatment responsive networks. The addition of rs-fMRI demonstrated that antidepressant response was associated with reduced fALFF in the SOFC [47]. A recent review summarized that the activity and connectivity in the prefrontal are the most consistent ECT antidepressant-response fMRI biomarkers [48]. The implications of reduced fALFF may also be consistent with the recent conceptualization of temporary disruption (reduced fALFF) followed by neuroplasticity and rewiring [49]. This model specifies that the optimal level of disruption and neuroplasticity will be associated with antidepressant response and cognitive safety.

ECT cognitive safety biomarkers are the neuroanatomic “anti-targets” for stimulation. This is the first data-driven investigation to identify the cognitive-impairment network associated with ECT. Changes in letter fluency were used to identify the cognitive impairment network. Letter fluency is a frontal-temporal cognitive function that is sensitive to ECT-associated cognitive impairment in adults across the lifespan [39, 50]. Relative to the antidepressant-response network, the cognitive-impairment network is dominated by decreased fALFF in the SOFC, caudate, hippocampus complex, and amygdala covaried with increased GMV in putamen and MTC. Preclinical investigations have demonstrated that electroconvulsive stimulations disrupt hippocampal long-term potentiation [51]. fALFF measures local spontaneous resting state neural activity reflecting energy metabolism and chemical signaling in the brain [52]. Therefore, reduced fALFF in the hippocampal complex may be consistent with disrupted long-term potentiation implicating this mechanism with ECT-mediated cognitive impairment. These results are consistent with a previous investigation that demonstrated that ECT-induced verbal dysfluency was associated with reduced hippocampal connectivity [53]. Thus, frontal-temporal networks may be vulnerable to ECT-associated disruption in long-term potentiation.

**Fig. 6:**
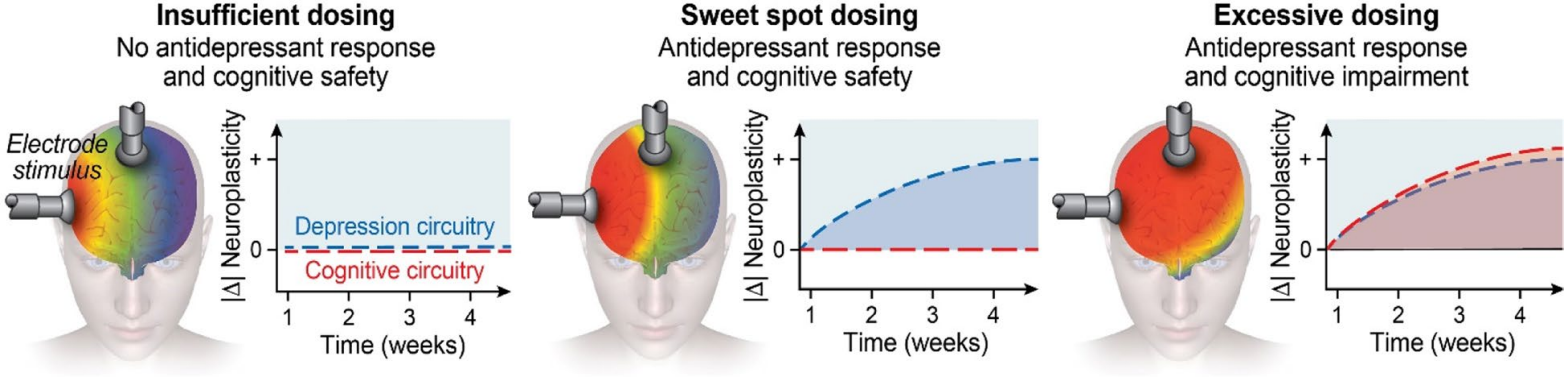
The relationship between E-field, neuroplasticity (structural and functional brain changes), and clinical outcomes. (Left) Insufficient E-field dosing with no changes in depression and cognitive related circuitry. (Middle) Optimal E-field dosing with increased grey matter volume and reduced fALFF in depression circuitry. (Right) Excessive E-field dosing with further reductions in fALFF in cognitive circuitry

The therapeutic and cognitive effects of ECT are dissociable [54]. This premise has also motivated the development of more focal methods of stimulus delivery, such as focal electrically administered seizure therapy (FEAST) and magnetic seizure therapy [55, 56]. The results of this study showed a high degree of overlap between antidepressant and cognitive networks, which motivates the development of more targeted methods of stimulus delivery designed to preserve antidepressant efficacy and maximize cognitive safety. Furthermore, the antidepressant and cognitive mechanisms may both utilize similar mechanisms. Neuroplasticity is a widely supported antidepressant mechanism but may also be implicated with remodeling and inefficient cognitive processing [57]. Interestingly, the hippocampal complex demonstrates both neuroplasticity (antidepressant-response network) and reduced fALFF (cognitive-impairment network). The E-field strength appears to mediate the inflection point between hippocampal neuroplasticity/antidepressant outcomes (higher is better) and hippocampal dysconnectivity/cognitive outcomes (lower is better). In addition, the E-field strength also mediates the potential therapeutic mechanism of “temporary disruption” (reduced fALFF associated with antidepressant benefit, higher is better) and disruptions in long-term potentiation of cognitive circuitry (reduced fALFF associated with cognitive impairment, lower is better). This conceptualization is consistent with the hypothesis that the distribution and strength of E-field are both associated with efficacy and cognitive side-effects [58]. Instead of focusing on the location of target engagement related to electrode placement and geometry of the E-field, we shift focus to amplitude and the relationship with E-field strength (Fig. 6). We found an optimal individualized pulse amplitude that will produce sufficient neuroplasticity and disruption of pathologic depression related circuitry (92.7 V/m) while avoiding excessive neuroplasticity and disruption of cognitive related circuity to promote cognitive safety (113.9 V/m). The estimation accuracy of pulse amplitude for cognition (AUC = 0.82) is higher than for antidepressant (AUC = 0.60), which means that the estimation for cognitive safety is more reliable than ECT response.

One limitation of this study is that the sample size is relatively small and there is no control group. However, in the context of single site, ECT multimodal imaging datasets, this is one of the largest datasets. In addition, the different amplitude arms (600, 700, and 800 mA) provide sufficient range in the induced E-field strength to allow for exploration of amplitude dose-dependent effects of ECT. Furthermore, independent ECT and MDD datasets validated the identified antidepressant-response and cognitive-impairment networks. Second, this study focused on an older adult sample of patients (50−80 years) treated with RUL electrode placement (with contingency switch to BT). Thus, the results may not be generalizable to younger adult populations and other traditional electrode configurations such as bifrontal placement. Third, although all antidepressant medications were discontinued, as needed medications were permitted with clear dose limitations to improve study feasibility. Fourth, the functional imaging parameters used in this study do not benefit from the advanced fMRI sequences which can increase spatial and temporal information. Note also, the duration of the scan (5min) is relatively short, and some studies have highlighted the need for longer scans, though the issue can be complicated due to issues related to patient comfort and changing connectivity patterns over longer durations. A possible solution going forward is to use spatially constrained ICA, which can obtain robust group differences and classification results with as little as 2–3 min of data [59]. Fifth, the voxel-wise GMV used in this study was generated from the basic voxel-based morphometry (VBM), not by the DARTEL that may improve the estimation of GMV. However, the majority of the previous published ECT sMRI analyses were based on VBM with increased hippocampus GMV after ECT is a consistent treatment response neuroimaging marker [17, 49]. Finally, the seizure was an essential therapeutic component of ECT and was not included in this investigation. We previously reported that the seizure duration was equivalent across all amplitude arms and have separately assessed the relationship between E-field strength and ictal EEG power [37, 60].

## Conclusions

In summary, to the best of our knowledge, this is the first study to identify the neurobiological underpinnings underlying ECT antidepressant-response and cognitive-impairment multimodal brain networks in late-life MDDs. We found large overlap between antidepressant and cognitive networks. The overlap in the hippocampal complex reflects different mechanisms with increased GMV in the antidepressant-response network and decreased fALFF in cognitive impairment network. Further investigation with the E-field associated with this trade-off between hippocampal GMV and fALFF changes may serve as potential biomarkers to balance antidepressant and cognitive safety with ECT. The determination of the optimal E-field within the hippocampal complex to balance GMV (antidepressant) and fALFF (cognitive) changes to estimate an optimal individualized amplitude, which determines the E-field strength, may improve the ECT benefit–risk ratio.

## Supplementary Information

**Additional file 1: Table S1.** Demographic and clinical information of 600, 700, 800 mA groups in discovery ECT1 cohort. **Table S2.** Anatomical information of the ECT antidepressant-response network. **Table S3.** Anatomical information of the identified cognitive impairment network. **Table S4.** Correlations between antidepressant and cognitive networks with ECT numbers. **Figure S1.** Linear projection of antidepressant network to an independent ECT dataset to test whether the ECT responsiveness of this network can be replicated. **Figure S2.** Linear projection of cognitive-impairment network to an independent ECT dataset to test whether the ECT responsiveness of this network can be replicated. **Figure S3.** Replication of antidepressant and cognitive impairment multimodal brain networks on RUL subset.

## Data Availability

The supervised fusion code has been released and integrated in the Fusion ICA Toolbox (FIT, https://trendscenter.org/software/FIT/), which can be downloaded and used directly by users worldwide. The ECT data and validation datasets used in the present study can be accessed upon request to the corresponding authors.

## Abbreviations

ECT: Electroconvulsive therapy
MDD: Major depressive disorder
E-field: Electric field
fALFF: Fractional amplitude of low frequency fluctuations
GMV: Gray matter volume
BT: Bitemporal
RUL: Right unilateral
sMRI: Structural MRI
SOFC: Superior orbitofrontal cortex
MTC: Medial temporal cortex
DKVFLFSS: Delis Kaplan Executive Function System Verbal Fluency Letter Fluency Total Correct Scaled Score
HDRS: Hamilton Depression Rating Scale
SimNIBS: Simulation of Non-Invasive Brain Stimulation
