# Correction: Links between electroconvulsive therapy responsive and cognitive impairment multimodal brain networks in late-life major depressive disorder

**DOI:** 10.1186/s12916-023-02800-2

**Published:** 2023-03-29

**Authors:** Shile Qi, Vince D. Calhoun, Daoqiang Zhang, Jeremy Miller, Zhi-De Deng, Katherine L. Narr, Yvette Sheline, Shawn M. McClintock, Rongtao Jiang, Xiao Yang, Joel Upston, Tom Jones, Jing Sui, Christopher C. Abbott

**Affiliations:** 1 College of Computer Science and Technology, Nanjing University of Aeronautics and Astronautics, Nanjing, China; 2 Tri-institutional Center for Translational Research in Neuroimaging and Data Science (TReNDS) Georgia State University, Georgia Institute of Technology, Emory University, Atlanta, GA USA; 3 Department of Psychiatry, University of New Mexico, Albuquerque, NM USA; 4 Noninvasive Neuromodulation Unit, Experimental Therapeutics & Pathophysiology Branch, National Institute of Mental Health, Bethesda, MD USA; 5 Departments of Neurology, Psychiatry and Biobehavioral Sciences, University of California, Los Angeles, CA USA; 6 Department of Psychiatry, University of Pennsylvania, Philadelphia, PA USA; 7 Division of Psychology, Department of Psychiatry, UT Southwestern Medical Center, Dallas, TX USA; 8 State Key Laboratory of Cognitive Neuroscience and Learning, Beijing Normal University, Beijing, China; 9 Huaxi Brain Research Center, West China Hospital of Sichuan University, Chengdu, China

**Correction: BMC Med 20, 477 (2022)**

**https://doi.org/10.1186/s12916-022-02678-6**

The original article [1] mistakenly omitted panel (g) from Figure 1; the panel has since been re-inserted.
