# Supplementary material for: Links between electroconvulsive therapy responsive and cognitive impairment multimodal brain networks in late-life major depressive disorder
Source: BMC Med. 2022 Dec 8;20:477. doi: 10.1186/s12916-022-02678-6 (PMC9733153; doi:10.1186/s12916-022-02678-6)
Supplement: Supplementary file 1 — Additional file 1: Table S1. Demographic and clinical information of 600, 700, 800 mA groups in discovery ECT1 cohort. Table S2. Anatomical information of the ECT antidepressant-response network. Table S3. Anatomical information of the identified cognitive impairment network. Table S4. Correlations between antidepressant and cognitive networks with ECT numbers. Figure S1. Linear projection of antidepressant network to an independent ECT dataset to test whether the ECT responsiveness of this network can be replicated. Figure S2. Linear projection of cognitive-impairment network to an independent ECT dataset to test whether the ECT responsiveness of this network can be replicated. Figure S3. Replication of antidepressant and cognitive impairment multimodal brain networks on RUL subset. [file 12916_2022_2678_MOESM1_ESM.docx]

**Links between Electroconvulsive Therapy Responsive and Cognitive Impairment Multimodal Brain Networks in Major Depressive Disorder**

**Running title:** Antidepressant and cognitive impairment networks

Shile Qi*^1^, Vince D. Calhoun^2^, Daoqiang Zhang^1^, Jeremy Miller^3^, Zhi-De Deng^4^, Katherine L. Narr^5^, Yvette Sheline^6^, Shawn M. McClintock^7^, Rongtao Jiang^8^, Xiao Yang^9^, Joel Upston^3^, Tom Jones^3^, Jing Sui*^8^, Christopher C. Abbott*^3^

1. College of Computer Science and Technology, Nanjing University of Aeronautics and Astronautics, Nanjing, China

Tri-institutional Center for Translational Research in Neuroimaging and Data Science (TReNDS) Georgia State University, Georgia Institute of Technology, Emory University, Atlanta, GA, USA

1. Department of Psychiatry, University of New Mexico, Albuquerque, NM, USA
2. Noninvasive Neuromodulation Unit, Experimental Therapeutics & Pathophysiology Branch, National Institute of Mental Health, Bethesda, MD, USA
3. Departments of Neurology, Psychiatry and Biobehavioral Sciences, University of California, Los Angeles, CA, USA
4. Department of Psychiatry, University of Pennsylvania, PA, USA
5. Division of Psychology, Department of Psychiatry, UT Southwestern Medical Center, Dallas, TX, USA
6. State Key Laboratory of Cognitive Neuroscience and Learning, Beijing Normal University, Beijing, China
7. Huaxi Brain Research Center, West China Hospital of Sichuan University, Chengdu, China

**Supplementary information**

# **Imaging parameters and preprocessing**

Exclusion criteria included neurological or neurodegenerative disorder (e.g., head injury, epilepsy, or Alzheimer’s disease), other psychiatric disorders (e.g., bipolar disorder, schizoaffective disorder, and schizophrenia), substance (except nicotine) or alcohol use disorder, and contraindications to MRI.

All MRI images were collected on UNM on a 3-Tesla Siemens Trio scanner. High-resolution T1-weighted structural images were acquired with a 5-echo MPRAGE sequence with TE = (1.64, 3.5, 5.36, 7.22, 9.08) ms, TR = 2.53 s, TI = 1.2 s, flip angle = 7, number of excitations = 1, slice thickness = 1 mm, field of view = 256 mm, resolution = 256 × 256. T2-weighted functional images were acquired with a gradient-echo EPI sequence with TE = 29 ms, TR = 2 s, flip angle = 75, slice thickness = 3.5 mm, slice gap = 1.05 mm, field of view 240 mm, matrix size = 64 × 64, voxel size = 3.75 × 3.75 × 4.55 mm. Resting state scans were acquired over a minimum of 5 min, 16 s in duration (158 volumes). Subjects were instructed to keep their eyes open during the scan and stare passively at a fixation cross.

For fMRI, standard preprocessing in SPM12 included the following: 1) realignment; 2) slice timing correction; 3) normalization to an EPI (3 $\times$ 3 $\times$ 3mm^3^) template; 4) spatial smoothing using a 6-mm full width half-maximum Gaussian kernel; 5) regression of parameters and nuisance variables (six parameters obtained by rigid body head motion correction, cerebrospinal fluid, white matter signals and global signal); and 6) calculation of fractional amplitude of low frequency fluctuations (fALFF).

The sMRI preprocessing included the following: 1) segmentation into gray matter (GM), white matter (WM) and cerebral spinal fluid (CSF); 2) normalization to Montreal Neurological Institute (MNI) space using the unified segmentation method in SPM12; 3) resliced to 3 $\times$ 3 $\times$ 3 mm^3^; and 4) smoothed with an full width-half maximum 6 mm Gaussian filter. After preprocessing, two representative MRI features (fALFF from fMRI and GM volume from sMRI) were extracted. Next, each modality containing the whole brain was reshaped into a feature matrix with columns representing voxels and rows representing subjects. Since $\Delta$KVFLFSS was correlated with number of major depressive episodes (**Table 1**), we regressed out number of major depressive episodes from fALFF and GM prior to fusion analysis. Finally, the obtained two feature matrices were normalized to have the same average sum of squares (computed across all subjects and all voxels for each modality) to ensure all modalities had the same range of values.

# **Head motion**

To control confounding effects of motion artifact, several strategies were conducted. We remove the outlier subjects who have micro motion such as frame-wise displacements (FD) exceeding 1 mm, as well as head motion exceeding 1.0 mm of maximal translation (in any direction of x, y or z) or 2.5^o^ of maximal rotation throughout the course of scanning. We further despiked the fMRI data, and regressed out six head motion parameters, white matter, and cerebrospinal fluid. Results indicate all FDs (mean framewise displacements, mean of root of mean square frame-to-frame head motions assuming 50 mm head radius) for all subjects were < 1 mm. We performed correlation analysis between clinical scores with mean FD, as displayed in **Table 1**. There is no significant correlations between the mean FD and$\Delta$HDRS/$\Delta$DKVFLFSS.

Furthermore, fMRI data were spatially smoothed with a 6 mm full width half max (FWHM) Gaussian filter. To calculate fractional amplitude of low frequency fluctuations (fALFF), the sum of the amplitude values in the 0.01 to 0.08Hz low-frequency power range was divided by the sum of the amplitudes over the entire detectable power spectrum (range: 0–0.25Hz). We believe micro-motion such as FD is not a major factor affecting the current results. Finally, the ICA-based fusion analysis was conducted on the spatial maps of fALFF not the function connectivity, which is impacted by head motion.

# **Education degree**

1 = grade 6 or less

2 = grade 7 -12 (without graduating high school)

3 = graduated high school

4 = part college or university

5 = graduated 2-year college (Associates Degree)

6 = graduated 4-year college (bachelor’s degree)

7 = part graduate or professional school

8 = completed graduate or professional school

# **Electric field**

Simulation of Non-Invasive Brain Stimulation (SimNIBS) creates subject specific, anatomically realistic volume conductor model. The T1 and T2-weighted scans are segmented into skin, bone, eyes, cerebral spinal fluid, ventricles, and grey and white matter with a combination of FMRIB Software Library (FSL) and Statistical Parametric Mapping 12 (SPM12) Computational Anatomy Toolbox. The E-field modeling used T1 and T2 structural scans. Earlier E-field modeling approaches used diffusion tensor imaging (DTI). However, a validation study demonstrated that white matter anisotropy did not improve E-field modeling accuracy and was therefore not included in this analysis. SimNIBS then turns this segmentation into a tetrahedral head mesh using GMSH, a three-dimensional finite element (FE) mesh generator, with unique conductivity values for each tissue type. Electrodes are added to the head mesh in either RUL or BT orientation and simulated with corresponding current. SimNIBS then uses a FE solver to calculate the voltages and electric fields corresponding to the stimulation throughout the head mesh. The initial E-field map is created with 1mA current placed that electrodes. We then use the input current as a multiplier to determine the final E-field strength (in our example: $\times$600, 700, or 800 mA) for subjects that completed the ECT series with RUL electrode placement. For subjects that required a BT electrode placement secondary to non-response to RUL at mid-series evaluation, we used the BT E-field geometry with 800 mA amplitude to calculate the E-field strength. To induce seizure activity, the ECT device delivers electrical pulses over an 8 second duration. The height of the pulse amplitude is related to electric field strength, and the geometry of this electric field is related to electrode placement (right unilateral or bitemporal). A typical pulse train will be 20 hertz over 8 seconds or 160 pulse pairs. The E-field model is the single pulse of one of these pulse pairs and influenced by individual anatomic differences in skull thickness, brain size, etc.

**Table S1.** Demographic and clinical information of 600, 700, 800 mA groups in discovery ECT1 cohort.

|  | **600 mA** | **700 mA** | **800 mA** | **ANOVA p-value** |
| --- | --- | --- | --- | --- |
| Demographic Characteristics |  |  |  |  |
| Sample size (*n*) | *n*=16 | *n*=19 | *n*=19 | n/a |
| Age (years) | 65.4$\pm$8.6 | 63.7$\pm$7.0 | 67.1$\pm$10.5 | 0.51 |
| Gender (M/F) | 4/12 | 4/15 | 8/11 | 0.34 |
| Ethnicity (Non-Hispanic/Hispanic) | 13/3 | 15/4 | 18/1 | 0.12 |
| Race (Caucasian/African American/Hispanic/Asian) | 14/0/1/1 | 15/0/4/0 | 17/1/1/0 | 0.56 |
| Education degree** | 5.5$\pm$1.8 | 5.3$\pm$2.1 | 5.4$\pm$1.6 | 0.96 |
| Handiness (R/L) | 16/0 | 19/0 | 19/0 | n/a |
| Height | 1.7$\pm$0.1 | 1.6$\pm$0.1 | 1.7$\pm$0.1 | 0.06 |
| Weight | 71.5$\pm$20.3 | 68.6$\pm$17.5 | 76.7$\pm$22.4 | 0.46 |
| BMI | 26.0$\pm$6.5 | 25.7$\pm$5.2 | 26.0$\pm$6.4 | 0.98 |
| IQ | 108.8$\pm$11.5 | 111.1$\pm$12.8 | 109.4$\pm$9.2 | 0.83 |
| Mean FD (PRE) | 0.2$\pm$0.1 | 0.2$\pm$0.1 | 0.3$\pm$0.1 | 0.65 |
| Mean FD (POST) | 0.2$\pm$0.1 | 0.07$\pm$0.2 | 0.3$\pm$0.1 | 0.47 |
| Clinical Characteristics |  |  |  |  |
| Age onset | 36.8$\pm$19.2 | 39.6$\pm$22.6 | 31.8$\pm$17.7 | 0.48 |
| Age treated | 41.5$\pm$16.1 | 45.5$\pm$17.9 | 34.5$\pm$17.4 | 0.15 |
| Single episode/recurrent | 1/15 | 3/16 | 2/17 | 0.32 |
| Number of major depressive episodes | 5.1$\pm$5.3 | 3.0$\pm$3.0 | 4.2$\pm$3.7 | 0.32 |
| Duration of current depressive episode (months) | 13.6$\pm$10.8 | 14.0$\pm$21.3 | 24.3$\pm$28.5 | 0.25 |
| Lifetime duration years (years) | 5.8$\pm$4.6 | 7.2$\pm$12.6 | 9.1$\pm$11.5 | 0.64 |
| Total number of ECT treatments | 10.4$\pm$2.9 | 10.7$\pm$3.7 | 11.2$\pm$3.3 | 0.78 |
| Maudsley scale for treatment resistance | 9.1$\pm$1.9 | 8.7$\pm$2.1 | 9.0$\pm$2.2 | 0.88 |
| Last treatment: RUL/BT | 10/6 | 13/6 | 10/9 | 0.62 |
| PRE HDRS | 37.3$\pm$8.3 | 37.9$\pm$6.6 | 33.4$\pm$6.8 | 0.13 |
| POST HDRS | 22.6$\pm$11.2 | 12.8$\pm$10.4 | 14.3$\pm$8.5 | 0.01 |
| ΔHDRS | 14.6$\pm$14.7 | 25.1$\pm$11.7 | 19.2$\pm$11.3 | 0.06 |
| PRE DKVFLFSS | 9.8$\pm$4.0 | 7.4$\pm$4.0 | 7.5$\pm$4.0 | 0.16 |
| POST DKVFLFSS | 7.2$\pm$3.9 | 5.0$\pm$3.2 | 5.4$\pm$4.4 | 0.25 |
| ΔDKVFLFSS | 0.8$\pm$3.4 | 2.7$\pm$2.0 | 2.3$\pm$3.0 | 0.14 |

# **Validation1: independent ECT dataset**

MDD patients (*n* = 84) were recruited from the University of New Mexico (UNM) and University of California Los Angeles (UCLA) after meeting the clinical indication for ECT. Two independent psychiatric examinations confirmed diagnosis prior to the initiation of ECT at both sites. Inclusion criteria included age (UNM: 50-80 years, UCLA: 18-75 years), treatment resistance (failure of two antidepressants), decisional capacity to consent to research (UNM and UCLA) or assent to research with surrogate decision maker consent (UNM). Exclusion criteria included the following: (1) defined neurodegenerative or neurological disorder (e.g., Alzheimer’s disease, epilepsy or head injury); (2) other psychiatric conditions (e.g., schizoaffective disorder, schizophrenia); (3) current alcohol or drug dependence; (4) pregnancy; and (5) contraindication to magnetic resonance imaging (MRI) (e.g., pacemaker). The clinical assessment was the 17-item Hamilton Depression Rating Scale (HDRS) at both sites.

MDD subjects completed the cognitive testing on the same date as MRI scans. Pre-ECT scans were completed within 2 days of ECT start and post-ECT assessment completed within 7 days of finishing ECT series. UCLA data completed a neuropsychological assessment that included the Hopkins Verbal Learning Test-Revised (HVLT-R). UNM data completed the Repeatable Battery of Neuropsychological Status (RBANS), which included a 10-word verbal learning and memory task. Percent recall for the HVLT-R and RBANS was calculated as the percentage of total words recalled during delayed recall trial relative to the maximum of the words from either the second or third learning trials. The percent retention score is a useful measure of hippocampal dependent memory function that reduces the possibility of over-estimating memory function from immediate and delayed free recall scores. ECT procedures were similar across both study sites. Subjects started with a right unilateral electrode placement unless bitemporal was clinically indicated (acuity, non-responsive to right unilateral electrode placement). The demographic and clinical characteristics of the sample are summarized in the following table.

T2-weighted functional images were acquired with a gradient-echo EPI sequence with TE = 29 ms, TR = 2 s, flip angle = 75, slice thickness = 3.5 mm, slice gap = 1.05 mm, field of view 240 mm, matrix size = 64 × 64, voxel size = 3.75 × 3.75 × 4.55 mm. Resting state scans were acquired over a minimum of 5 min, 16 s in duration (158 volumes). Subjects were instructed to keep their eyes open during the scan and stare passively at a fixation cross. Structural magnetic resonance imaging data were collected at UNM on a 3 Tesla Siemens Trio scanner (repetition time=2.53 s (s), echo time=1.64, 3.50, 5.32, 7.22, 9.08 ms, inversion time=1.20 s, flip angle=7, number of excitations=1, and voxel size=1 × 1 × 1 mm3), LIJ on a 3T GE HDx scanner, and UCLA on a 3-T Allegra scanner. The same preprocessing procedures were done with fMRI and sMRI to generate fALFF and GMV.

Demographic and clinical information of an independent validation1 dataset.

|  | **MDD** | ***p*1#** | ***p*2##** |
| --- | --- | --- | --- |
| Demographic Characteristics |  |  |  |
| Sample size (*n*) | *n*=84 | n/a | n/a |
| Age (years) (mean/sd) | 52.3$\pm$16.4 | 4.1e-04 | 0.18 |
| Gender (M/F) | 34/50 | 0.17 | 0.54 |
| Education degree** (mean/sd) | 5.3$\pm$2.0 | 0.80 | 0.25 |
| Handiness (R/L) | 77/7 | 0.96 | 0.10 |
| Mean FD (PRE) | 0.14$\pm$0.18 | 0.10 | 0.36 |
| Mean FD (POST) | 0.20$\pm$0.16 | 0.09 | 0.88 |
| Clinical Characteristics |  |  |  |
| Number of major depressive episodes | 9.6$\pm$21.4 | 0.87 | 0.63 |
| Duration of current depressive episode (months) | 26.7$\pm$37.9 | 0.17 | 0.57 |
| Total number of ECT treatments | 10.6$\pm$3.5 | 0.94 | 0.07 |
| RUL/mixed RUL-bitemporal, and BT* | 63/21 | 0.06 | 0.95 |
| $\Delta“\% recall”$ of verbal declarative memory | 10.5$\pm$72.0 | 0 | 0.77 |
| $\Delta$HDRS | 13.4$\pm$10.4 | 0.77 | 0 |

* RUL: Right unilateral, BT: bitemporal.

**“Education degree” details are presented in **Additional file 1:** “**Education degree**” section.

# “*p*1” denotes the *p* values for the correlation between $\Delta$HDRS.

## “*p*2” denotes the *p* values for the correlation between $\Delta“\% recall”$.

A$\pm$B represents mean $\pm$ standard deviation.

Δ means PRE-POST.

**Validation2: independent baseline MDD dataset**

Independent baseline MDDs (*n* = 260, age: 32.8 ± 11.0, 99 male) were recruited from the West China Hospital of Sichuan. Both resting state fMRI and sMRI data were collected and were preprocessed to fALFF and GMV using the same processing pipeline. Note that HDRS is available for all the *n*=260 MDDs, cognition (verbal fluency) is only available for *n*=48 MDDs.

The resting state fMRI data were collected on a 3T whole MR scanner (Achieva, Philips, Netherlands) using an eight-channel phased-array head coil. During scanning, foam padding and earplugs were used to minimize the head movement and scanner noise. All participants underwent a high resolution three-dimensional T1-weighted, sagittal, magnetization-prepared rapid gradient echo (MPRAGE) sequence with the following parameters: repetition time (TR) = 8.4 ms; echo time (TE) = 3.9 ms; flip angle = 7°; in-plane matrix resolution = 256$\times$256; field of view (FOV) = 256$\times$256 mm; voxel size = 1$\times$1$\times$1 mm^3^; thickness = 1 mm; number of slices = 188. A total of 240 volumes of echo planar images were obtained axially with a gradient echo EPI sequence with the following parameters: TR = 2000 ms; TE = 30 ms; in-plane matrix resolution = 64$\times$64; field of view = 240$\times$240 mm; number of slices = 38. For the resting scan, subjects were instructed to lie still with eyes closed.

# **Linear projection**

In this study, we further tested the replicability of the identified ECT antidepressant and cognitive-impairment networks, *i.e.*, whether the ECT responsiveness (longitudinal differentiating between PRE and POST ECT) extracted in discovery ECT dataset can be validated in independent ECT dataset, by performing cross-site linear projection analysis. Starting with fMRI, after ICA decomposition, $\boldsymbol{S}_{\mathrm{fMRI}}^{ECT1}\boldsymbol{=}\left( \boldsymbol{A}_{\mathrm{fMRI}}^{ECT1} \right)^{-}\boldsymbol{X}_{\mathrm{fMRI}}^{ECT1}$ was generated in the discovery cohort (ECT1). For the validation cohort (ECT2), we obtained the mixing matrix by linear projection as: $\boldsymbol{A}_{\mathrm{fMRI}}^{ECT2}=\boldsymbol{X}_{\mathrm{fMRI}}^{ECT2}\times\left( \boldsymbol{S}_{\mathrm{fMRI}}^{ECT1} \right)^{-}$ ($\boldsymbol{S}_{\mathrm{fMRI}}^{ECT1}$ from ECT1 was used as spatial maps for ECT2). The same approach was used for sMRI projection from ECT1 to ECT2. Paired T-test was used to calculate the longitudinal difference of the generated loadings (the same index in ECT1, *i.e.*, IC1) between PRE and POST ECT. Correlation analysis was performed between loadings and HDRS/cognition.

# **Power analysis**

With respect to the power analysis, here we provide an example of calculating the statistical power of longitudinal HDRS change by using G*Power software (<http://www.softpedia.com/get/Science-CAD/G-Power.shtml>). As in our study, the sample size is 54 MDDs. The effect size of HDRS change for differentiating between PRE and POST ECT (*p* = 1.2e-15) was computed using Cohen's *d* = 2.2, according to equation (1). Given the significance level $\alpha$ = 0.05, sample size (54 MDDs), and the effect size = 2.2, the statistical power of the longitudinal difference of HDRS is 1. The same method was used to calculate the longitudinal difference of loadings (**Fig. 2**), achieving the statistical power of 0.99 for fALFF_IC1, and 1 for GM_IC1, respectively, which are all high enough to assure accurate and robust conclusions with regard to the longitudinal effects detected in our current study.

$d=\frac{M_{1}-M_{2}}{\sqrt{\left( \sigma_{1}^{2}+\sigma_{2}^{2} \right)/2}}$ (1)

where $M_{1}$ is the mean of PRE ECT group and $M_{2}$ is the mean of the POST ECT group. $\sigma_{1}$ and $\sigma_{2}$ represent the standard deviation of PRE and POST groups respectively.

**Table S2.** Anatomical information of the **ECT antidepressant-response network**.

| **fALFF_IC1 Area** | **Brodmann Area** | **volume (cc)**  **R/L** | **random effects: Max Value (x, y, z) R/L** |
| --- | --- | --- | --- |
| **Negative** |  |  |  |
| Superior/Middle Temporal Gyrus | 19, 21, 22, 37, 38,39,42 | 2.8/2.7 | 8.7 (-65, -34, 16)/7.8 (59, -55, 17) |
| Superior/Middle Frontal Gyrus | 9, 10, 11, 46, 47 | 2.0/1.8 | 4.8 (-45, 46, -5)/6.3 (24, 52, -8) |
| Parahippocampus/Hippocampus/Amygdala | 34 | 1.3/0.3 | 5.5 (-15, -7, -15)/4.8 (33, -13, -17) |
| Caudate |  | 0.1/0.1 | 2.3 (-9, 11, -3)/2.2 (48, 14, -3) |
| **GM_IC1 Area** | **Brodmann Area** | **volume (cc)**  **R/L** | **random effects: Max Value (x, y, z)**  **R/L** |
| **Positive** |  |  |  |
| Thalamus |  | 0.9/2.5 | 2.9 (-12, -29, 10)/3.8 (15, -26, 10) |
| Superior/Middle Temporal Gyrus | 13, 19, 21, 22, 37, 39, 41 | 2.7/0.8 | 3.5 (-50, -37, 16)/3.1 (53, -43, 21) |
| Parahippocampus/Hippocampus | 19 | 1.0/0.5 | 3.4 (-30, -38, -3)/2.1 (21, -35, 5) |
| Insula | 13 | 1.4/0.6 | 3.2 (-42, -37, 18)/3.1 (50, -40, 21) |
| Caudate |  | 0.1/0.0 | 2.2 (-15, 4, 14)/NaN |

**Table S3.** Anatomical information of the identified **cognitive impairment network**.

| **fALFF_IC1 Area** | **Brodmann Area** | **volume (cc) R/L** | **random effects: Max Value (x, y, z) R/L** |
| --- | --- | --- | --- |
| **Negative** |  |  |  |
| Parahippocampus/Hippocampus/Amygdala | 28, 34, 36 | 1.2/2.0 | 5.8 (-15, -7, -15)/10.3 (15, -10, -17) |
| Medial Frontal Gyrus | 10, 11, 25 | 0.5/1.0 | 3.0 (-9, 34, -14)/8.1 (15, 58, -5) |
| Caudate |  | 0.1/0.0 | 2.4 (-9, 11, -3)/-999.0 (0, 0, 0) |
| **GM_IC1 Area** | **Brodmann Area** | **volume (cc) R/L** | **random effects: Max Value (x, y, z) R/L** |
| **Positive** |  |  |  |
| Middle Temporal Gyrus | 21, 22, 37, 39 | 3.2/1.2 | 4.3 (-50, -53, -2)/2.8 (45, -70, 6) |
| Insula | 13 | 1.9/0.8 | 3.6 (-45, -37, 21)/2.8 (48, -40, 21) |

# **Effect of ECT number**

We calculated the correlation between ECT treatment number and the brain imaging features (fALFF and GM) before fusion analysis. Results showed that there was no association between ECT number and brain imaging features for PRE- or POST-ECT (*p*<1.0e-04, not FDR corrected for multiple comparisons). We also calculated the correlation between the identified brain networks with ECT treatment number. The results showed no significant correlations between the identified ECT responsive and cognitive networks with ECT session number, as displayed in the following **Additional file 1: Table S4**.

**Table S4.** Correlations between antidepressant and cognitive networks with ECT numbers.

| **Treatment responsive network** | ***r*** | ***p*** |
| --- | --- | --- |
| **fALFF_IC1** |  |  |
| Corr(Pre-ECT loading coefficient, ECT number) | 4.6e-05 | 0.99 |
| Corr(Post-ECT loading coefficient, ECT number) | 0.03 | 0.78 |
| **GM_IC1** |  |  |
| Corr(Pre-ECT loading coefficient, ECT number) | 0.11 | 0.41 |
| Corr(Post-ECT loading coefficient, ECT number) | -0.03 | 0.80 |
| **Cognitive impairment network** | ***r*** | ***p*** |
| **fALFF_IC1** |  |  |
| Corr(Pre-ECT loading coefficient, ECT number) | -0.01 | 0.93 |
| Corr(Post-ECT loading coefficient, ECT number) | 0.16 | 0.22 |
| **GM_IC1** |  |  |
| Corr(Pre-ECT loading coefficient, ECT number) | -0.06 | 0.65 |
| Corr(Post-ECT loading coefficient, ECT number) | 0.16 | 0.23 |


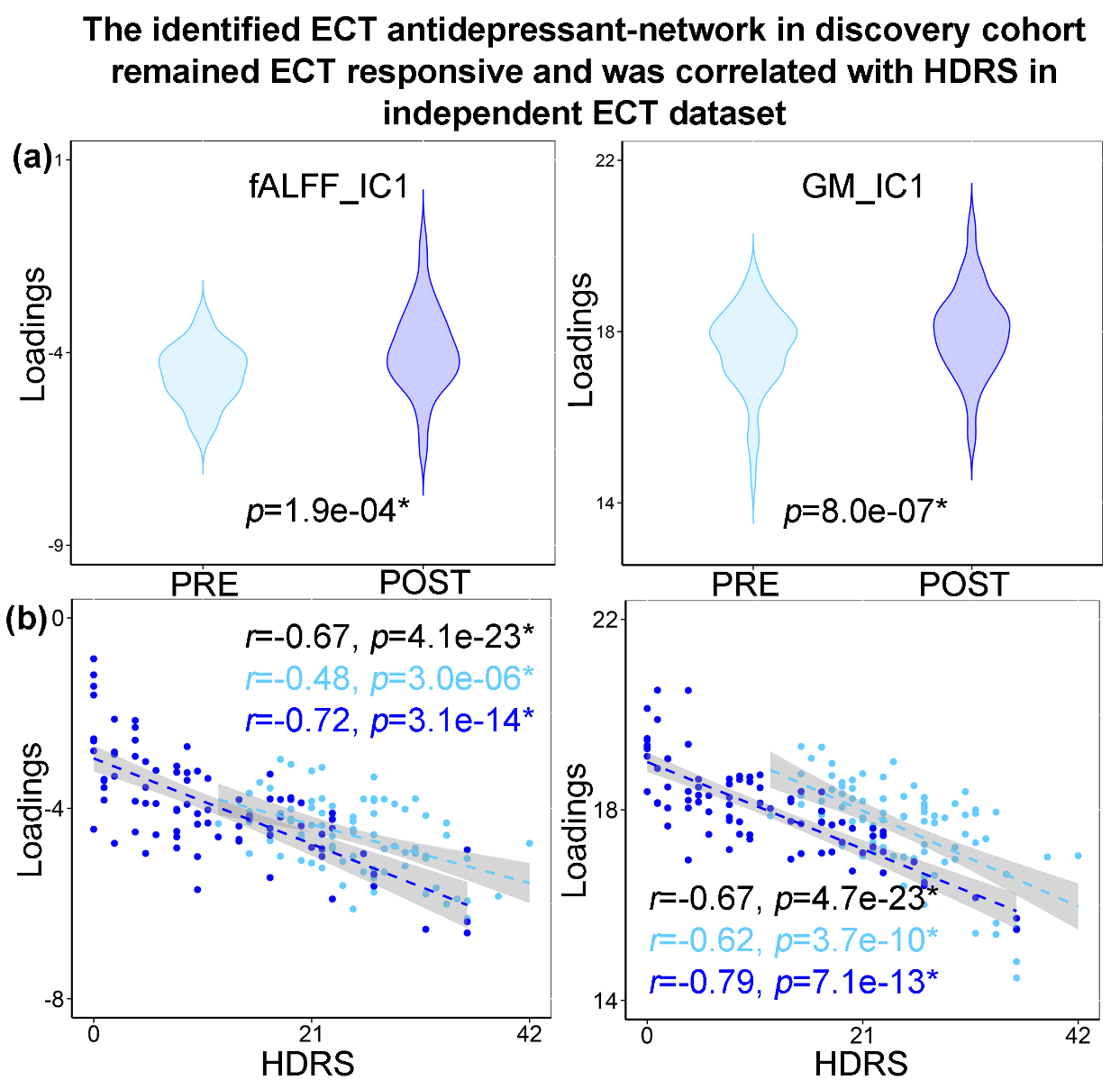


**Figure S1.** Linear projection of antidepressant network to an independent ECT dataset to test whether the ECT responsiveness of this network can be replicated. (b) Longitudinal PRE and POST ECT difference of the projected loadings in independent ECT dataset. (c) Correlation between projected components’ loadings and HDRS.


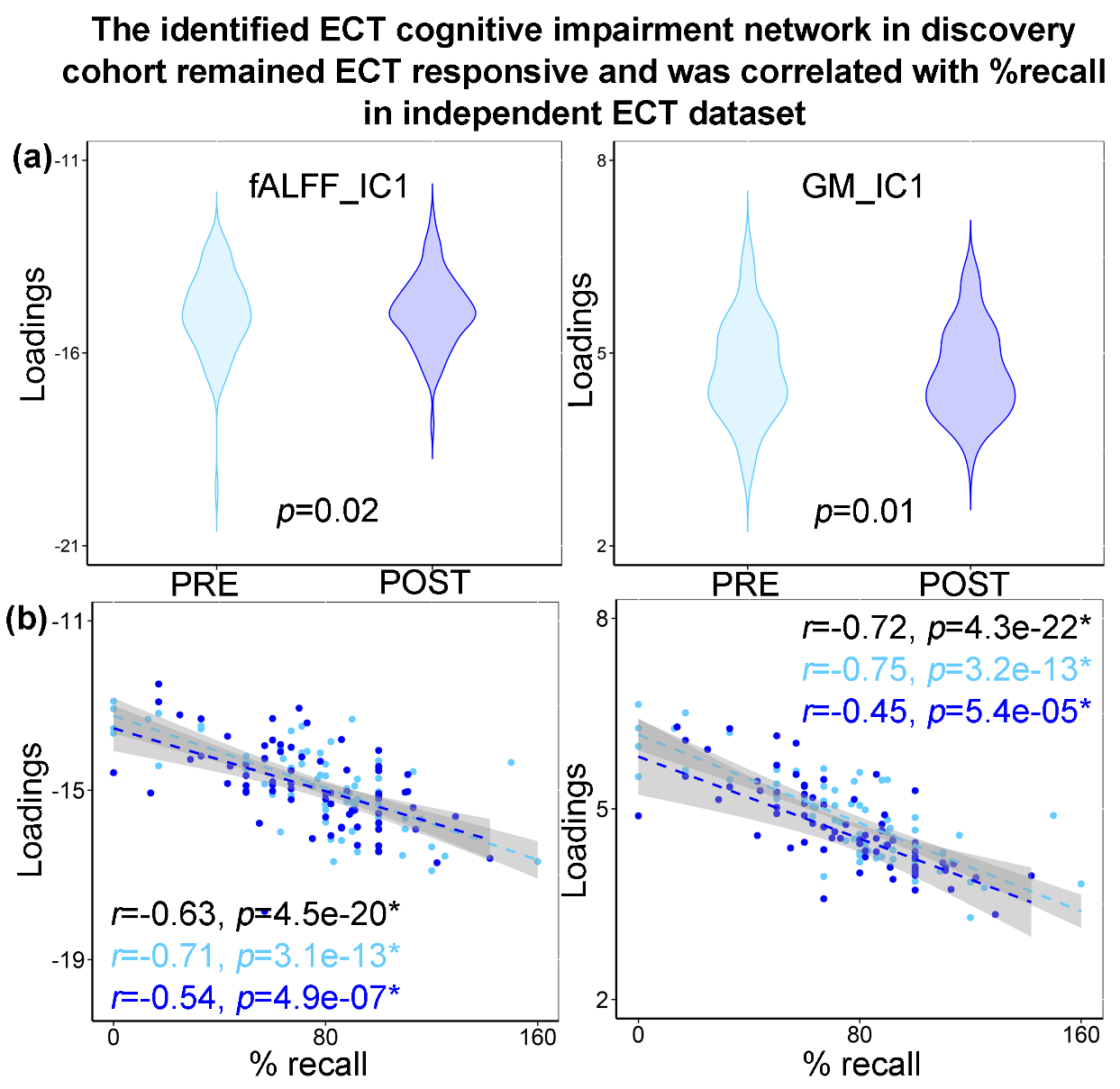


**Figure S2.** Linear projection of cognitive-impairment network to an independent ECT dataset to test whether the ECT responsiveness of this network can be replicated. (b) Longitudinal PRE and POST ECT difference of the projected loadings in independent ECT dataset. (c) Correlation between projected components’ loadings and % recall.

# **RUL only**

The HDRS/DKVFLFSS guided fusion were performed on RUL subset to see whether the identified antidepressant and cognitive impairment networks can be replicated. It is clear that the all the identified common and difference between antidepressant and cognitive impairment networks can be replicated on RUL only subset (*n*=33), as displayed in **Additional file 1: Fig. S3c-d**.

**
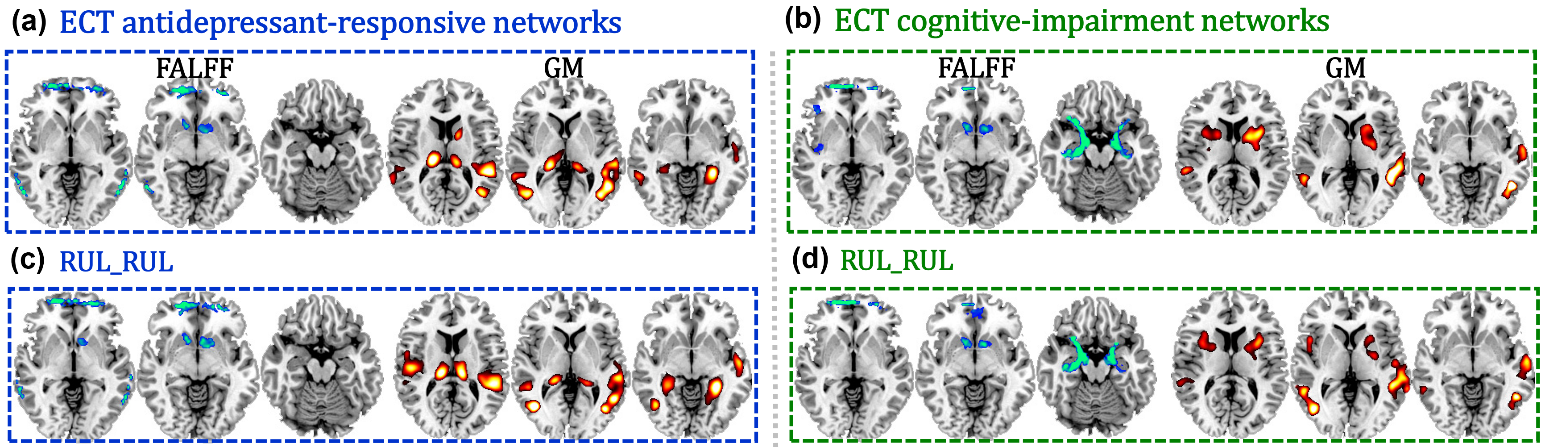
**

**Figure S3.** Replication of antidepressant and cognitive impairment multimodal brain networks on RUL subset. RUL_RUL represent start with RUL and end with RUL for ECT treatment.

The common areas were identified by overlapping fALFF/GMV component between antidepressant and cognitive-impairment networks. Results showed that decreased fALFF in the superior orbitofrontal cortex and caudate accompanied with increased GMV in medial temporal cortex showed covarying functional and structural alterations in both antidepressant-response and cognitive-impairment networks. Normalized average fALFF/GMV were computed within these common areas (ROIs, superior orbitofrontal cortex and caudate in fMRI and medial temporal cortex in sMRI). Correlation between common areas and HDRS/cognition were calculated. Note that HDRS is available for all the *n*=260 MDDs, cognition (verbal fluency) is only available for *n*=48 MDDs.
